# Unraveling the dynamic mechanisms of natural killer cells in viral infections: insights and implications

**DOI:** 10.1186/s12985-024-02287-0

**Published:** 2024-01-12

**Authors:** Arash Letafati, Omid Salahi Ardekani, Mina Naderisemiromi, Mehdi Norouzi, Mohammadreza Shafiei, Soheil Nik, Sayed-Hamidreza Mozhgani

**Affiliations:** 1 Department of Virology, Faculty of Public Health, Tehran University of Medical Sciences, Tehran, Iran; 2 Research Center for Clinical Virology, Tehran University of Medical Science, Tehran, Iran; 3 Department of Immunology, Faculty of Medicine and Health, The University of Manchester, Manchester, UK; 4 School of Medicine, Alborz University of Medical Sciences, Karaj, Alborz Iran; 5 Department of Microbiology and Virology, School of Medicine, Alborz University of Medical Sciences, Karaj, Iran

**Keywords:** Natural killer cells, Antiviral immunity, Innate immune response, Viral infections, NK cell alteration, Chronic infection

## Abstract

Viruses pose a constant threat to human well-being, necessitating the immune system to develop robust defenses. Natural killer (NK) cells, which play a crucial role in the immune system, have become recognized as vital participants in protecting the body against viral infections. These remarkable innate immune cells possess the unique ability to directly recognize and eliminate infected cells, thereby contributing to the early control and containment of viral pathogens. However, recent research has uncovered an intriguing phenomenon: the alteration of NK cells during viral infections. In addition to their well-established role in antiviral defense, NK cells undergo dynamic changes in their phenotype, function, and regulatory mechanisms upon encountering viral pathogens. These alterations can significantly impact the effectiveness of NK cell responses during viral infections. This review explores the multifaceted role of NK cells in antiviral immunity, highlighting their conventional effector functions as well as the emerging concept of NK cell alteration in the context of viral infections. Understanding the intricate interplay between NK cells and viral infections is crucial for advancing our knowledge of antiviral immune responses and could offer valuable information for the creation of innovative therapeutic approaches to combat viral diseases.

## Introduction

Viral infections continue to pose an enduring challenge to human health, giving rise to a wide spectrum of illnesses that vary in their severity and can even result in fatal outcomes. Indeed, recent assessments have underscored the noteworthy influence of viral infections on the overall burden of cancer across the globe, accounting for roughly 10% of the total cancer burden worldwide. The outcome of an infection heavily relies on the intricate interaction between viruses and the host immune system. Natural killer (NK) cells, among the various cellular elements of the immune system, have been recognized as crucial participants in the protection against viral infections. These remarkable innate immune cells possess the unique ability to directly recognize and eliminate infected cells, making them crucial in the early control and containment of viral pathogens [1, 2].

NK cells, classified as innate lymphocytes, play a pivotal role as an initial defense mechanism against both tumors and viral infections. The increased susceptibility to viral diseases observed in people with congenital NK cell deficiency emphasizes the importance of NK cells in the immune response against viruses [3]. Humans have two major subsets of NK cells (CD56^bright^CD16^low/−^ and CD56^dim^CD16^+^), which have different functions in immunity. CD56^bright^ cells play a more immunomodulatory role and can generate significant quantities of cytokines. These cells possess limited capacity to eliminate targeted cells while CD56^dim^ cells serve as cytotoxic effectors and it has the ability to cause lysis in target cells [4]. NK cells are finely tuned to recognize and react to viral infections through a complex interplay of activating and inhibitory receptors. These receptors allow NK cells to differentiate between healthy cells and those infected by viruses. The activation status and subsequent response of NK cells depend on the interplay between activating and inhibitory signals [5, 6]. When NK cells are activated, they deploy various mechanisms to eliminate cells infected by viruses. One of their main methods involves directly eliminating targeted cells by releasing cytotoxic granules that contain perforin and granzymes. Perforin creates pores in the target cell membrane, allowing granzymes to enter and initiate the process of apoptosis. Additionally, NK cells have the capability to initiate the death of target cells by interacting with death receptors through the Fas ligand (FasL) or tumor necrosis factor-related apoptosis-inducing ligand (TRAIL) [7, 8]. Apart from their cytotoxic effects, NK cells play a significant role in the antiviral defense by releasing a diverse array of proinflammatory cytokines that possess antiviral properties [9]. Moreover, NK cells can induce apoptosis by binding to antibodies that have opsonized infected cells, using the CD16 receptor. This mechanism, referred to as antibody-dependent cellular cytotoxicity (ADCC) [10]. These effector functions collectively contribute to the elimination of infected cells and limit viral spread.

However, emerging research has shed light on an intriguing phenomenon observed during viral infections: the alteration of NK cells [11–13]. Besides their well-established role in antiviral defense, NK cells can undergo dynamic changes in their phenotype, function, and regulatory mechanisms upon encountering viral pathogens. These alterations can significantly impact the effectiveness of NK cell responses during viral infections. In this review, we delve into the multifaceted role of NK cells in antiviral immunity, exploring both their conventional effector functions and the emerging concept of NK cell alteration in the context of viral infections.

## An overview of NK cell characteristics

### NK cell development

**Fig. 1:**
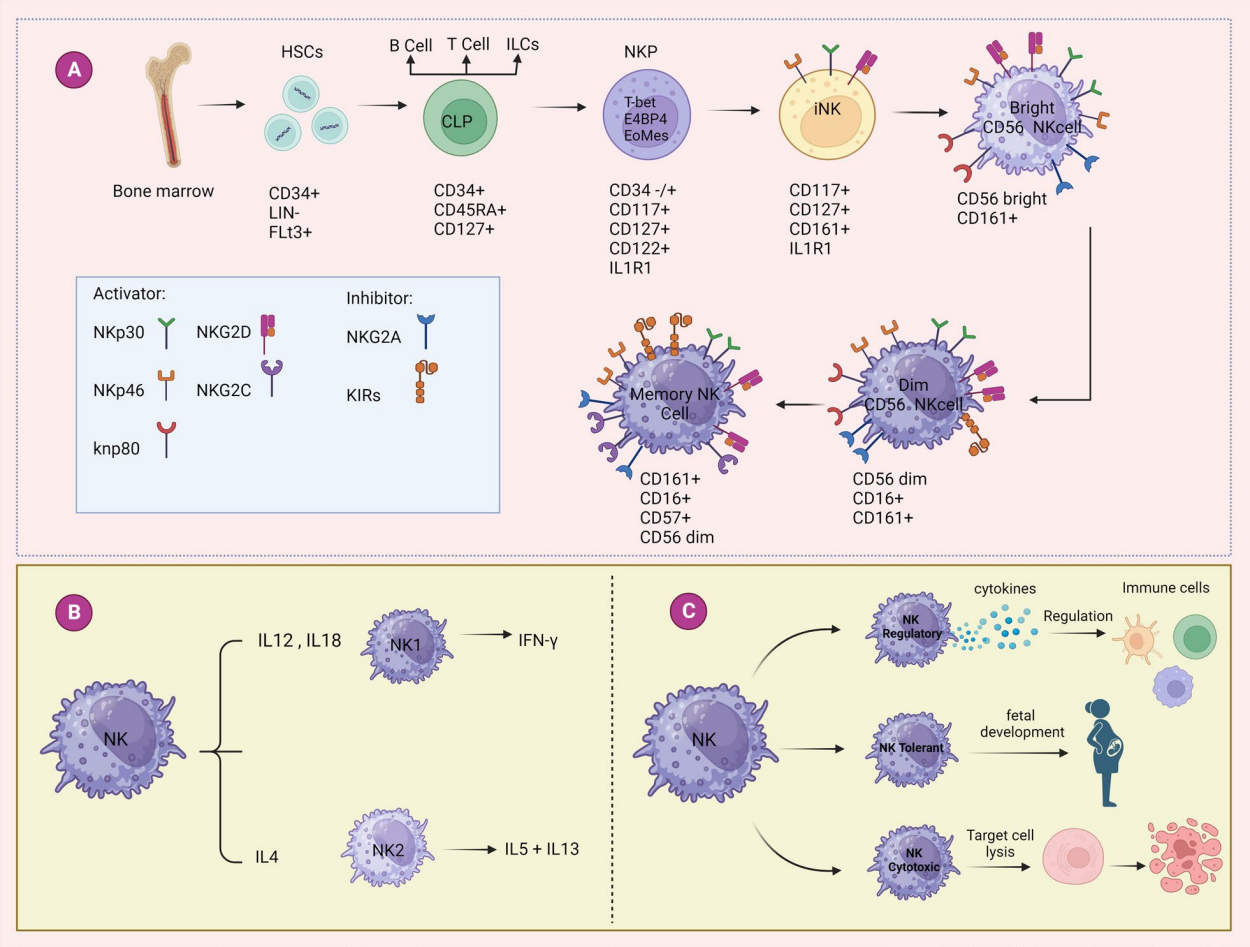
**A** Developmental pathway of NK cells. The developmental pathway of NK cells from the bone marrow involves an ongoing process of differentiation and maturation. It begins with hematopoietic stem cells (HSCs) residing in the bone marrow, which serve as the origin for NK cell development. Firstly, HSCs differentiate into Lymphoid-primed Multipotent Progenitors (LMPPs). Subsequently, LMPPs give rise to Common Lymphoid Progenitors (CLPs), which possess the ability to develop into various lymphoid lineages, including NK cells. Following the emergence of NK cell progenitors from CLPs, the developmental process continues onward. Subsequently, these NK cell progenitors undergo further differentiation, resulting in the formation of mature NK cells. These mature NK cells are identifiable by their expression of CD56 and CD16. **B** In vitro, human NK cells can differentiate into two distinct functional subsets known as NK1 and NK2. When cultured with IL-12 and IL-18, NK cells (NK1) primarily generate IFN-γ, while those cultured with IL-4 (NK2) predominantly produce IL-5 and IL-13. **C** Human NK cells can be categorized into three distinct functional subsets based on their roles, specifically referred to as NK tolerant, NK cytotoxic, and NK regulatory. Figure created using BioRender (Created with biorender.com)

NK cells are vital elements of the innate immune system and have a significant function in safeguarding the host from viral infections and malignant cells. These unique lymphocytes are originated from the common lymphoid progenitors (CLPs) in the bone marrow (BM) and undergo a complex developmental process to acquire their effector functions. However, emerging evidence has suggested that their development is not narrow to the BM but also occurs in secondary lymphoid tissues likes spleen, tonsils, and lymph nodes [14]. NK cell development begins with the commitment of hematopoietic progenitor cells towards the NK cell lineage (Fig. 1A).

In the beginning stages, hematopoietic stem cells (HSCs) lacking the Lin marker but expressing CD34, CD133, and CD244 undergo differentiation into a specific subset known as lymphoid-primed multipotential progenitors (LMPPs) that are CD45RA, CD133 and CD34 positive. Then, these cells undergo differentiation into a subset known as CLPs that are CD7, CD10, CD34, CD38, CD127 and CD45RA-positive. CLPs, known for their multipotency, have the capability to differentiate into diverse lineages. These lineages encompass Pro-B cells, Pre-T cells, progenitors of natural killer cells (NKPs), and innate lymphoid cells (ILCs). The presence of IL-15/IL-2 receptor common β-chain (CD122) expression, provide compelling evidence of the commitment of CLP cells to the development of the NK cell lineage [15, 16]. Instead of a particular transcription factor, a diverse set of transcription factors has been discovered to play vital roles in the development and maturation of NK cells. These transcription factors, namely T-bet, GATA-2, EOMES, E4BP4, ID2, RUNX3, and BLIMP, work together in a coordinated manner to orchestrate the intricate process of NK cell development [17]. After this, the emergence of CD56 (neural cell adhesion molecule or NCAM) expression signifies the conclusive transformation of immature NK cells (iNK) into their mature counterparts. The majority of iNK cells undergo a shift towards a minor population characterized by CD56^bright^, which subsequently transitions into the predominant CD56^dim^ population. As well, there is a suggested possibility that iNK cells have the potential to directly generate the CD56^dim^ population. It is worthy to note that, the development and homeostasis of NK cells heavily rely on the presence of common gamma chain cytokines, namely IL-2, IL-7, and IL-15, and their corresponding receptor components, such as CD122 and CD127 [15, 16]. New findings have presented a new existing model, indicating the potential for a more complex pattern of development involving both CLPs and CMPs, resulting in the emergence of NK cell progenitors. The revised pattern also suggests that separate precursor populations undergo independent maturation processes leading to different types of mature NK subsets [18].

### NK cell subsets

Approximately 15% of lymphocytes in humans are NK cells, characterized by their lack of CD3 expression and presence of CD56. These cells can be categorized into two subsets: CD56 ^bright^ and CD56^dim^, each with unique phenotypic features based on their level of CD56 expression on the cell surface. Most of the NK cells found in humans are characterized as CD56^dim^ and exhibit elevated expression of CD16 (FcγRIII). While the remaining are CD56^bright^ CD16^dim^ or CD56^bright^CD16^−^ [19]. The cells known as CD56^bright^ are mainly found in secondary lymphoid tissues, whereas CD56^dim^ cells are primarily found in peripheral blood (PB) [20]. Moreover, the human NK cells characterized into three distinct subsets based on their functions, namely NK^tolerant^, NK^cytotoxic^, and NK^regulatory^ (Fig. 1C). Cytotoxic NK cells, characterized by the CD56^dim^ CD16^bright^ phenotype, primarily engage in cell lysis as their primary function. Regulatory NK cells play a role in modulating the functions of other immune cells, including dendritic cells (DCs), macrophages, and T cells. Tolerant NK cells represent a passive subset of regulatory cells that play a crucial role in maintaining self-tolerance. Additionally, tolerant NK cells are recognized for their capacity to induce vascular remodeling, providing physiological support for fetal development [21, 22]. CD56^dim^ NK cells demonstrate significantly greater cytotoxic activity compared to CD56^bright^ cells due to their abundance of perforin, granzymes, and cytolytic granules [23]. Additionally, the significant amount of CD16 expressed on CD56^dim^ cells allows them for effective mediation of ADCC. On the other hand, CD56^bright^ cells exhibit weak ADCC. However, CD56^bright^ NK cells are recognized as highly efficient cytokine producers, notably releasing key cytokines including interferon-γ (IFN-γ), as well as tumor necrosis factor-α (TNF-α), granulocyte–macrophage colony-stimulating factor (GM-CSF), IL-10 and IL-13. Due to their ability to produce diverse cytokines, they can potentially play crucial roles in the early immune responses, influencing the development of adaptive responses, and functioning as regulatory NK cells [24]. Also, it is worthy to hint that, CD56^bright^ cells are the only ones that express c-kit receptor and the high-affinity receptor for IL-2, and they proliferate when exposed to low levels of this cytokine. On the other hand, CD56^dim^ NK cells display distinct characteristics compared to their CD56^bright^ counterparts. These cells primarily express the IL-2Rβγ, while lacking the expression of c-kit, leading to limited response to high concentrations of IL-2 [19]. Moreover, there is a unique cell named, Natural killer T (NKT) that exhibit characteristics of both the innate and adaptive immune systems. They possess markers of NK cells, along with T-cell receptor (TCR) α/β. Unlike regular T-lymphocytes, NKT cells do not engage in TCR-mediated interactions with peptide antigens presented by MHC class I or II molecules. Rather, their TCR specifically detects glycolipids that are displayed by CD1d, a non-classical molecule involved in presenting antigens [25]. NKT cells that are activated have the ability to generate a diversity of cytokines with both pro and anti-inflammatory effects. These cytokines include IFN-γ, TNF-α, IL-2, IL-4, IL-17, and tumor growth factor β (TGF-β) [26]. Interestingly, in contrast to conventional NK cells, which are short-lived innate lymphocytes with no antigen specificity, recent research has unveiled a distinct group of mature NK cells that possess an enduring “adaptive” quality, particularly in the specific setting of cytomegalovirus (CMV) infection. The presence of NKG2C and killer immunoglobulin-like receptors (KIR) can be observed in these cells, which are acknowledged for their high frequency characteristics [27]. These cells may primarily develop in the liver as evidenced by the expression of liver-specific adhesion molecules, namely CXCR6 and CD49a. Early indications of memory NK cells were first observed in a murine cytomegalovirus infection (MCMV). In this study, murine NK cells, exhibited quick reactions to a subsequent exposure to the same haptens through the activation of Ly49H^+^ receptors, which detect the virally-encoded m157 on infected cells [28]. Recent research indicates that adaptive NKG2C^+^ NK cells demonstrate differential recognition of various human cytomegalovirus infection (HCMV) strains carrying diverse UL40 peptides. Consequently, it is suggested that, the variability observed in adaptive NKG2C + NK cell populations in HCMV-seropositive individuals is attributed to the presence of diverse HCMV peptides with polymorphic characteristics [29]. Additionally, it has been revealed that memory NK cells offer protective benefits in the context of other viruses like herpes simplex virus 2 (HSV-2) and vaccinia virus infection models, as well as simian immunodeficiency virus (SIV) infection [30–32]. Remarkably, Research has demonstrated that human NK cells have the capacity to differentiate in vitro into two distinct functional subsets known as NK1 and NK2 (Fig. 1B). These subsets exhibit specific patterns of cytokine secretion, resembling the characteristics observed in Th1 and Th2 cells. NK cells that were cultured with IL-12, known as NK1 cells, are capable of producing IL-10 and IFN-γ. Moreover, these NK1 cells exhibit the ability to suppress IgE production. In contrast, when NK cells are cultured in the presence of IL-4, known as NK2 cells, they produce IL-5 and IL-13, which in turn stimulate IgE production [33–35]. Despite the similarity in cytotoxic activity between these NK cell subsets, NK1 cells exhibit elevated degrees of cell surface CD95 (Fas) antigen compared to NK2 cells. As a result, NK1 cells demonstrate increased sensitivity to apoptosis induced by antibodies or chemical agents [33]. NK2 cells, through the secretion of Th2 cytokines, may have a connection with allergic conditions such as asthma, which accounts for a substantial portion of asthma-related fatalities. Cytokines produced by NK2 cells, including IL-4 and IL-13, could potentially exacerbate asthma by supporting Th2 responses and inhibiting IFN responses, which may increase the likelihood of asthma worsening triggered by viral infections [36]. Respiratory Syncytial Virus (RSV) has been characterized as evading the host’s antiviral defenses by promoting immune responses skewed towards Th2 cells. One notable consequence of RSV infection is the activation of mast cells, which can be triggered by RSV binding to their IgE receptors, hypoxic conditions, or direct infection. Mast cell activation leads to the generation of chemotactic substances like CCL4 and histamine, which attract NK cells to the site of infection. Within the Th2-predominant environment found in the lungs during RSV infection, the recruited NK cells are likely to undergo differentiation into a subset known as NK2 cells. It is plausible that these recruited NK cells, now differentiated as NK2 cells, will produce cytokines including IL-4, IL-5, and IL-13 and lower antiviral cytokine IFN-γ, leading to asthma exacerbations [36].

### NK cell receptors

Function of NK cells depend on an equilibrium between their activating and inhibitory receptors (Fig. 1A). The natural cytotoxicity receptors (NCRs) and NKG2D are the primary receptors that activates NK cells and participating in NK cell-mediated lysis. The NCRs, which are a type I transmembrane molecule and belong to the immunoglobulin-like family, are constituted of three elements: NKp46 (NCR l), NKp44 (NCR2), and NKp30 (NCR3). NCRs are receptors that participate in NK-mediated cytolysis by identifying ligands that are distinct from MHC class I [37]. While NKp46 and NKp30 are present on almost all resting human NK cells and their levels are increased on stimulated NK cells, NKp44 is exclusively expressed on CD56^bright^ NK cells in a constant manner. However, after cytokine activation, almost all NK cells obtain the expression of NKp44 [6]. The NKG2D receptor, classified as a type II transmembrane and C-type lectin-like receptor, stands out from other members of the NKG2 family because, unlike them, it does not interact with CD94. This receptor is constantly present on the surface of all human NK cells. Its ligands are the molecules related to MHC class I, specifically MICA and MICB, and the UL16-binding protein (ULBP) of the human cytomegalovirus [38]. Additionally, CD16 (FcγRIII) serves as an activating receptor found on the surface of NK cells, which engages in ADCC by binding to the IgG [39]. NK cells have a close interaction with adaptive immunity and contribute to effectiveness of specific therapeutic monoclonal antibodies (mAbs) through this mechanism (ADCC) [40]. NKG2C is another receptor that, like NKG2A, attaches to HLA-E, albeit with less affinity and with activating effect [37]. Furthermore, NK cells have two primary types of inhibitory receptors that identify human leukocyte antigen (HLA) molecules. The first type is known as killer immunoglobulin-like receptors (KIR) and can identify HLA-A, HLA-B, or HLA-C allotypes including KIR2DL (except KIR2DL4 which have activating effect that identify HLA-G) and KIR3DL [41]. However, some types of KIRs activate NK cells and have short cytoplasmic tails that correlate with the DAP12 signaling adapter including KIR2DS and KIR3DS1 [41]. The second type is CD94/NKG2A, a heterodimer that recognizes HLA-E. The immunoreceptor Tyr-based inhibitory motif (ITIM) is utilized by both these types of inhibitory receptors to transmit signals [42]. Moreover, there are several co-receptors, namely 2B4, DNAM-1, CD59, and NKp80, that play a role in enhancing activating signals. These co-receptors work in tandem with other activating receptors on the surface of NK cells to amplify the immune response [6, 37, 38]. CD56^bright^ NK cells exhibit minimal expression of KIRs, but demonstrate elevated expression of CD94/NKG2A inhibitory receptors. Conversely, CD56^dim^ NK cells have high levels of KIR expression and lower expression of CD94/NKG2A inhibitory receptors [19]. The migration of CD56^bright^ NK cells to secondary lymph nodes is facilitated by the adhesion molecule L-selectin. This molecule, along with CC-chemokine receptor 7 (CCR7), contribute to this process. Comparison of CD56^dim^ and CD56^bright^ NK Cells are summarized in (Table 1) [19, 23, 24].

**Table 1:** Comparison between CD56^dim^ NK Cells and CD56^bright^ NK Cells

Feature	CD56^dim^ NK Cells	CD56^bright^ NK Cells
CD56 expression	Low	High
CD16 expression	High	Low/negative
ADCC	High	Low
Cytotoxicity	More cytotoxic	Less cytotoxic
Cytokine production	Lower cytokine production	Higher cytokine production
Surface marker expression	High KIR, and low to negative CD94/NKG2A	Low to negative KIR and high CD94/NKG2A
CD117 (c-Kit)	Negative	Positive
CCR7	Negative	Positive
CXCR3	High	Low to negative
CXCR1/CX3CR1	Positive	Negative
L-selectin (CD62L)	Low to negative	High
NKp46	Lower	Higher
Localization	Predominantly peripheral tissues	Predominantly secondary lymphoid tissues
Developmental stage	More mature	Less mature
Proliferation rate in response to IL-2	Low	High

ADCC, Ab-dependent cellular cytotoxicity; CCR7, CC-chemokine receptor 7; KIR, killer cell Ig-like receptor; CXCR, CX-chemokine receptor; CX3CR1, CX3C-chemokine receptor 1

## NK cell in virus infection

### Modes of target recognition by NK cells and subsequent activation

**Fig. 2:**
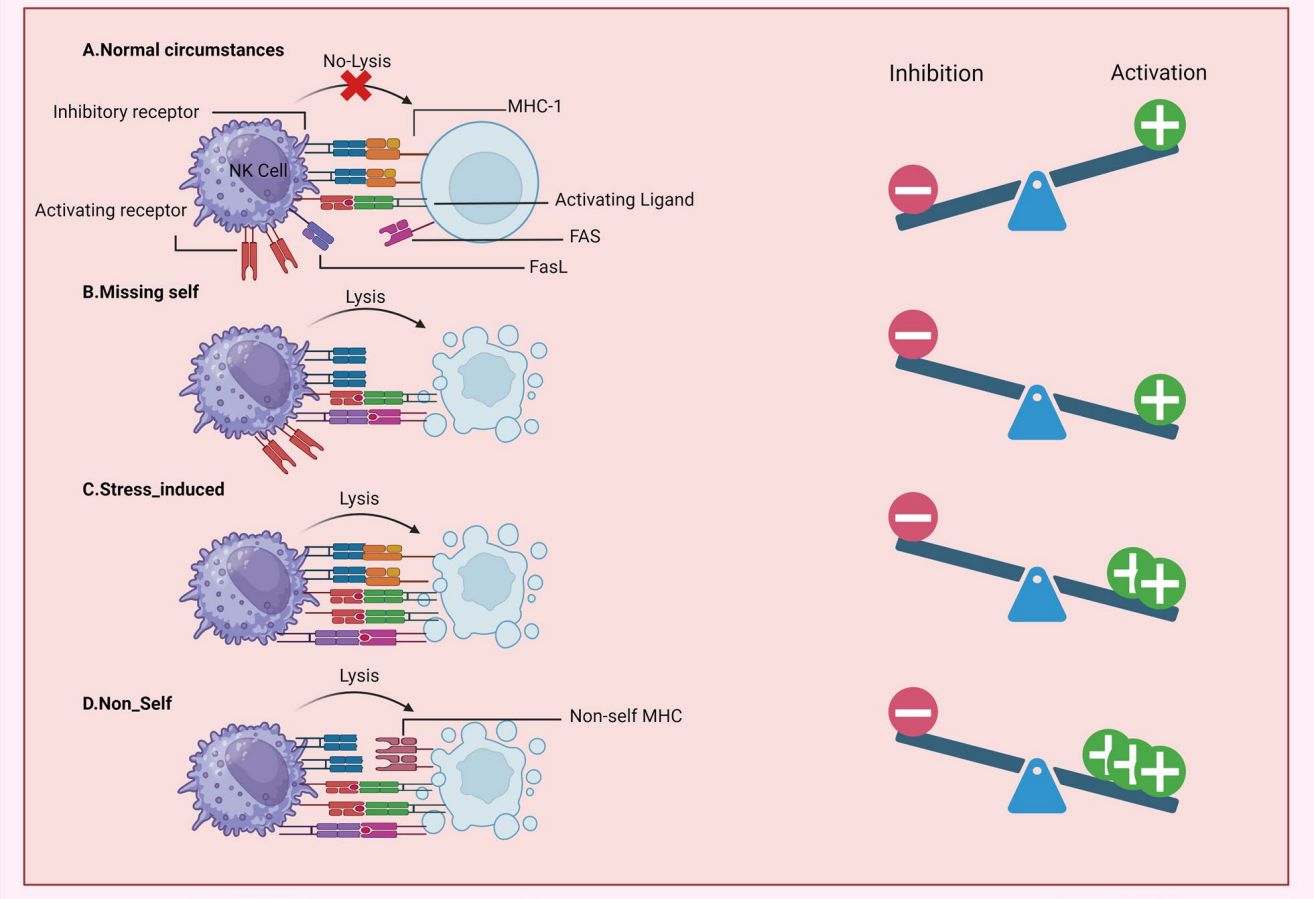
Mechanisms of target recognition by NK cells and subsequent activation. Their activation is tightly regulated and involves a complex interplay of activating and inhibitory signals. “Normal circumstance”: NK cells recognize autologous MHC class I molecules through inhibitory receptors like killer cell immunoglobulin-like receptor (KIR), indicating the interaction with normal cells and suppressing their activation. “Missing-self”: NK cells recognize target cells that either lack expression or have reduced levels of MHC class I molecules, such as infected or transformed cells. This recognition leads to the activation of NK cells. “Stress-induced”: NK cells recognize activating ligands expressed on target cells through NK receptors like NKG2D. This recognition can override MHC class I-mediated inhibitory signaling, resulting in the activation of NK cells. “Non-self”: NK cells recognize transplanted tissue when the donor tissue expresses either allogeneic or haploidentical MHC class I molecules. Figure created using BioRender (Created with biorender.com/)

NK cell activation refers to the procedure by which NK cells, are stimulated to exert their immune functions. NK cells have a significant role in the innate immune response against viral infections and malignant cells. Their activation is strongly regulated and involves a complex interplay of activating and inhibitory signals. The inhibitory receptors like KIRs identify MHC class I molecules expressed on normal cells [37, 38]. Under normal circumstances, when an NK cell encounters a healthy cell expressing sufficient levels of MHC class I molecules, the inhibitory receptors on the NK cell recognize these molecules and transmit inhibitory signals that prevent the activation of the NK cell. In this scenario, NK cells encounter both activating and inhibitory signals, but the inhibitory signals prevail, preventing the NK cell from being activated [15]. However, when an NK cell encounters a target cell that lacks or has reduced expression of MHC class I molecules, such as infected or transformed cells, the inhibitory signals are diminished or absent. This absence of inhibitory signals leads to a shift in the balance toward activation, resulting in NK cell activation (Missing-self) (Fig. 2) [43, 44]. Apart from the inhibitory signals, NK cells also have activating receptors that detect particular ligands present on target cells. When these activating receptors (like NKG2D & NCRs) interact with their corresponding ligands, a chain of internal signaling events are commenced, ultimately leading to the activation of the NK cell. The activation of the NK cell occurs when the target cell undergoes stress, possibly caused by an underlying infection. This stress prompts the production of activating ligands such as MIC-A/B within the target cell, ultimately leading to the overall activation of the NK cell. NK cell activation can also occur when the target cell exhibits non-self MHC-I molecules, such as in the case of an allogeneic transplant [5, 15, 45].

### NK cell activation during viral infection and its mechanisms of action

**Fig. 3:**
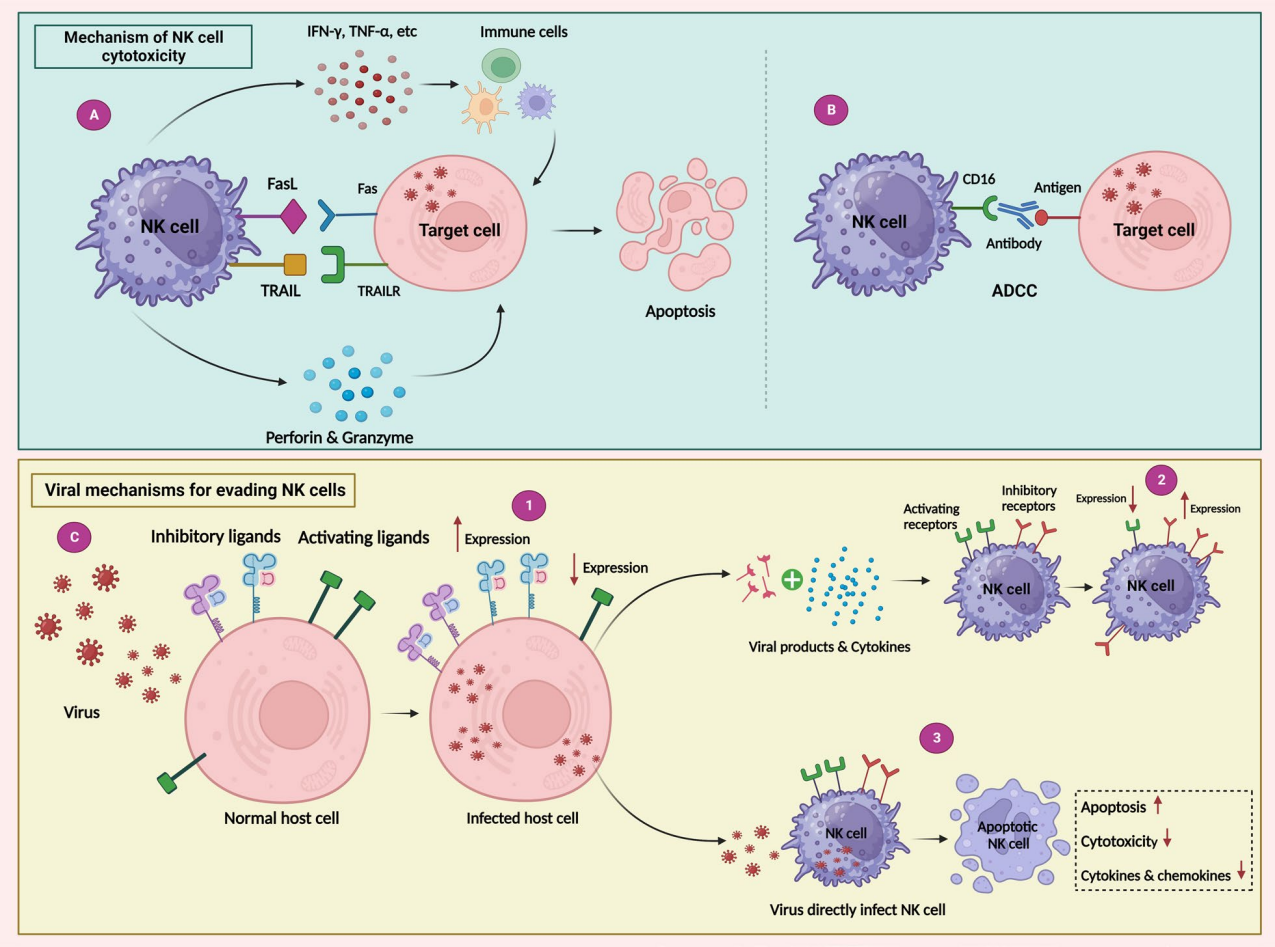
**A**, **B** NK cells mechanism of actions for cytolytic purpose. NK cells employ multiple mechanisms of action to exert their cytolytic function against target cells. These mechanisms involve a coordinated interplay of activating and inhibitory signals, leading to target cell recognition, activation, and destruction. The primary mechanisms of NK cell-mediated cytotoxicity include: (1) Release of Cytotoxic Granules, (2) Death Receptor Pathway, and (3) ADCC. **C** Viral mechanisms for evading NK cell: (1) Elevating the levels of inhibitory ligands (including HLA-E and HLA-C) and decreasing the presence of activating ligands (including ULBP1, ULBP2, MIC-A MIC-B) on the infected cell. (2) Viral products or cytokines released from infected cells have the ability to alter the quantity of receptors on NK cells. This alteration involves an increase in the expression of inhibitory receptors such as NKG2A and a decrease in the expression of activating receptors such as NKG2D on the infected cell. (3) Several viruses have the ability to directly disrupt the functioning of NK cells by infecting them. Viral infection can induce immune suppression, either by suppressing the cytotoxic function of NK cells or by initiating apoptosis, resulting in the depletion of NK cells

NK cells are the primary subsets of innate lymphocytes responsible for facilitating immune responses against both tumors and viral infections. Patients with a congenital deficiency in NK cells demonstrate heightened vulnerability to viral diseases, emphasizing the significance of NK cells in the immune response against viruses [3]. These cells play a crucial role in the initial immune response to viral infections, specifically by eliminating cells that have been infected by the virus. Different modes of NK cell activation can occur in the context of a viral infection (Fig. 3A, B). Initially, when a virus infects the cells, certain cytokines like type I IFN and IL-12 are generated as a response. The effector functions of NK cells are greatly enhanced by these cytokines. NK cells possess receptors for these cytokines, and their expression is notably increased shortly after infection. These receptors play a vital role in activating the NK cell population and facilitating protection against the virus [45]. Moreover, when a cell becomes infected by a virus, it often experiences a lessening in the expression of MHC I molecules on its surface. This alteration makes the infected cell more vulnerable to the NK cells. NK cells possess the remarkable capability to recognize and target cells with diminished MHC I expression through activating receptors [46]. For instance, the p12^I^ protein, which is encoded by Human T-lymphotropic virus 1 (HTLV-1) ORF-I, can be found in the endoplasmic reticulum (ER) and Golgi and have the ability to bind to the human MHC-I heavy chains. This interaction averts the correlation of β2-microglobulin with the newly synthesized MHC-I and causes it to be retrotranslocated to the cytosol for degradation by the proteasome complex. This process ultimately leads to reduced expression of MHC-I at the cell membrane and, as a result, enables evasion from cytotoxic T lymphocytes (CTLs) [47]. This mechanism (MHC-1 downregulation) is seen in many of viruses like Adenovirus, HCMV, Kaposi’s sarcoma-associated herpesvirus (KSHV), human immunodeficiency virus (HIV-1) [46] and also Influenza A and B viruses [48]. While this enables the virus to hinder the host cell’s ability to display viral protein-derived peptides to virus-specific CTLs, it also increases the susceptibility of the infected cell to NK. Moreover, the receptors present on NK cells that enable activation are responsible for triggering the activation process when they detect viral or stress-related molecules on the surface of target cells. To give an example, the engagement between NKp46, NKp44, NKp30, and the hemagglutinin (HA) protein of the Influenza virus is a well-documented process through which NK cells identify and eliminate targeted cells [49]. Additionally, NKp30 and NKp46 can detect the HA protein of poxviruses [50]. The hemagglutinin-neuraminidase found in avian Newcastle disease virus and human parainfluenza virus 3 (HPIV3) acts as the ligand for NKp44 and NKp46 [51, 52]. Furthermore, the activation of NK cells occurs when they bind to target cells that have been opsonized by antibodies, specifically through the interaction between Fc-γ receptor III (CD16) and these antibodies. This binding triggers a process known as ADCC (Fig. 3B). This mechanism has been proposed to play a part in various viral infections such as influenza and HIV infections [53, 54]. In addition, it is important to mention that NK cells have the ability to express toll-like receptors (TLRs). When these receptors come into contact with bacterial or viral substances and are accompanied by pro-inflammatory cytokines, they have the capacity to trigger a robust activation of NK cells [55]. Upon NK cell activation subsequent to a viral infection, a delicate equilibrium of inhibitory and activating receptors allows these cells to identify the altered cells and initiate a strong immune response, resulting in the destruction of the virus-infected cells. Once an infected cell is identified, NK cells have the aptitude to eliminate virally infected cells via two mechanisms: the targeted release of lytic granules comprising perforin and granzymes or by initiating death receptor-mediated apoptosis by expressing Fas ligand or TRAIL (Fig. 3A) [8]. Apart from their cytotoxic effects, NK cells play a significant role in the antiviral defense by releasing a diverse array of proinflammatory cytokines that possess antiviral properties [9]. These substances play a vital role in directly combating the virus, attracting other immune cells towards the location of the infection, and influencing the immune response in a specific direction. By means of these mechanisms, NK cells possess distinct capabilities to identify and promptly react to viral infections. The function of NK cells is affected by various cytokines, like IL-2, IL-12, IL-15, IL-18, and type I interferons. These cytokines can be generated by cells that are infected with viruses or antigen-presenting cells (APCs) that have been activated. The presence of these cytokines, supports the survival, growth, cytotoxicity, and cytokine secretion of NK cells, including the production of IFN-γ [56]. Interestingly, unique genetic connections exist between KIRs on NK cells and their specific HLA haplotypes, which in turn impact viral infections. One noteworthy instance is when individuals infected with HIV possess both KIR3DS1 and the HLA-Bw4-I80 allele, resulting in a favorable outcome. This combination is connected with a protective effect, resulting in reduced viral load and a slower onset of acquired immunodeficiency syndrome (AIDS) progression [57].

### Viral evasion from NK cell

In order to counteract the immune response mounted by NK cells, viruses have evolved and adopted diverse mechanisms and strategies to evade elimination (Fig. 3C). These strategies allow viruses to evade the surveillance and attack of NK cells, enabling them to establish persistent infections within the host. One way to evade elimination by NK cells is by reducing the levels of NK cell activator ligands. For instance, HIV-1 virus diminishes the presence of MICA, ULBP1, and ULBP2 (referred to as NKG2D ligands) on the surface of infected cells. This decrease is caused by the Nef protein, which in turn lowers the virus’s vulnerability to being destroyed by NK cells [58]. The ORF54 gene-encoded protein produced by KSHV reduces the expression of NKp44 ligand on infected cells to avoid being destroyed by NK cells [59]. Additionally, HCMV degrades the level of NKG2D ligands on infected cells by producing viral glycoproteins UL142 [60]. Another interesting approach is used by HTLV-1 to evade NK cell lysis. It has revealed that NK cells are incapable of eliminating primary CD4^+^ T cells infected with HTLV-1 in vitro. One contributing factor to this deficiency is the reduced capability of NK cells to attach to HTLV-1-infected cells, as a result of the HTLV-1 p12^I^ protein causing a decline in the levels of intercellular adhesion molecules 1 and 2 (ICAM-1 and ICAM-2). Furthermore, it is discovered that HTLV-1 infected CD4^+^ T-cells did not produce the ligands required for activating receptors in NK cells such as NCR and NKG2D but expressed ligands for NK cell co-activating receptors [61]. A further way to evade NK cell antiviral activity is to directly infecting them by the virus. It is evident that various viruses have the ability to directly disrupt the normal functioning of NK cells by infecting them. Viral infections can result in immune suppression by either reducing the cytotoxic activity or inducing apoptosis, which ultimately leads to diminution in the number of NK cells. One example of this approach is Influenza A virus (IAV). The process of IAV infecting NK cells involves clathrin- and caveolin-dependent endocytosis, enabling viral entry. In vitro trials reported that, IAV infection leads to reduced cytotoxic activity and triggers apoptosis in NK cells [62, 63]. The Epstein Barr virus (EBV) primarily targets lymphocytes and has the ability to infect NK cells by binding to CD21 receptors. It is intriguing to hint that NK cells acquire CD21 receptors upon interaction with CD21-positive cells infected by the EBV [64, 65]. Studies conducted in vitro have reported changes in the shape and size of NK cells following EBV infection, with infected cells exhibiting deformities and increased size. Additionally, in vivo studies have shown a correlation between EBV infection and the advancement of malignant conditions in NK cells, signifying that the virus plays a role in transforming these cells [66–68]. HIV-1 is an additional virus capable of infecting NK cells. The infection occurs through CD4, although not typically found in the NK lineage, can be upregulated in NK cells upon infection with human herpesvirus 6 (HHV-6). This upregulation enables subsequent HIV infection and leads to NK cell apoptosis [69, 70]. HTLV-1, unlike HIV, has a limited ability to infect CD4^+^ T-cells through cell-free transmission. It relies on cell-to-cell interactions for successful infection. When a cell that has been infected makes contact with an uninfected cell, a specific region called the virological synapse is formed. This synapse is created when a microtubule-organizing center (MTOC) becomes polarized at the junction between the cells. At the synapse, the HTLV-1 Gag complex and viral genomic RNAs gather and transfer into the uninfected cell. The involvement of ICAM1 enhances the polarization of the MTOC at the site of contact, highlighting the importance of the interaction between ICAM1 and lymphocyte function-associated antigen 1 (LFA1) in HTLV-1 infection. Not only T cells, the primary targets for HTLV-1, but also CD4^+^ T cells and NK cells have been observed to exhibit this transfer mechanism. However, the transmission of HTLV-1 from T cells to NK cells necessitates the activation of NK cells through the utilization of an anti-FcγRIII antibody. Infection of NK cells with HTLV-1 promotes the proliferation and survival of these cells. Although HTLV-1-infected NK cells produce viral proteins, they do not generate infectious viral particles. Nonetheless, they retain their capacity to facilitate both natural and antibody-dependent cell cytotoxicity [71–73]. Furthermore, the Herpesviridae family encompasses two additional viruses, namely herpes simplex virus (HSV) and varicella zoster virus (VZV), which can infect NK cells through cell–cell interactions. HSV has been observed to enter NK cells when they are in close proximity and co-cultured with infected fibroblasts. Similarly, recent in vitro findings have exhibited the infection of NK cells by VZV after co-culture with infected epithelial cells [74, 75]. When NK cells are exposed to VZV (varicella-zoster virus) in a laboratory setting, it results in a reduction in the levels of CD56 and FcγRIII on the surface of NK cells. However, there is a concurrent increase in the expression of CD57, which is considered a characteristic feature of mature NK cells. Moreover, VZV infection triggers the activation of C–C chemokine receptor type 4 (CCR4) and cutaneous lymphocyte antigen (CLA) on NK cells that have been infected. This activation leads to the development of a characteristic phenotype that promotes the migration of these cells towards the skin [75]. It is possible that RSV can enter NK cells through a process called macropinocytosis. This infection of NK cells by RSV leads to an augmented production of IFN-γ, a potent cytokine involved in immune response. Simultaneously, the expression of inhibitory KIRs is upregulated. Additionally, even in the absence of infection, the presence of RSV or RSV-antibody complexes leads to reduced levels of expression of the activating receptors NKG2D and NKp44 on NK cells [76, 77]. In addition to these viruses, there are several other viruses that have the capability to infect NK cells, each leading to distinct outcomes.

Moreover, chronic infection-inducing viruses have a unique strategy in addition to the mentioned evasion mechanisms. They directly hinder the cytotoxicity of NK cells by modifying their phenotypes and functions. Furthermore, certain chronic viral infections have the potential to trigger a state of NK cell exhaustion like HBV, HCV and HIV [57, 78, 79]. These viruses can employ multiple strategies to evade or disrupt NK cell function, which could potentially play a part in the persistence of the virus.

For instance, in the case of chronic Hepatitis C virus (HCV) infection, NK cells exhibit changes in their phenotypes and functions. Most people who are infected with HCV experience a long-lasting infection that leads to ongoing inflammation and liver diseases [80]. One reason why HCV is able to establish a persistent infection is because it can evade the immune response via various means, including the suppression of NK cells. During the initial phase of HCV infection, NK cells play a crucial role in controlling viral replication and influencing the progression to chronic disease. They achieve this by producing IFN-γ, which helps restrict the replication of the virus in hepatocytes and initiates adaptive immune responses [81]. However, in individuals with chronic HCV infection, the function of NK cells is noticeably compromised. Several investigations have been organized to assess the characteristics and activities of NK cells in patients with HCV, yielding varying and inconclusive findings. However, the majority of these studies have consistently reported a compromised functionality of NK cells in HCV infection. In chronic HCV infection, NKG2D expression on circulating NK cells is reduced due to the action of NS5A. This reduction in NKG2D expression results in impaired NK cell-mediated cytotoxicity and IFN-γ generation. NS5A stimulates monocytes through Toll-like Receptor 4 (TLR4), leading to the production of IL-10 in a p38- and PI3 kinase-dependent manner. At the same time, it inhibits the generation of IL-12. The presence of IL-10 induces the secretion of TGF-β, which further decreases NKG2D expression on NK cells [82]. It has been indicated that the continuous rise in NKG2A expression in NK cells within the liver also plays a significant role in causing NK cell exhaustion. This exhaustion ultimately leads to the persistence of HCV in the body. The infection caused by HCV also leads to the upregulation of Qa-1 expression in hepatocytes. This upregulated Qa-1 binds to NKG2A, triggering NK cell exhaustion [79]. A study, revealed that, NKp30 expression is suppressed in the context of chronic HCV infection. This suppression is achieved by elevating the levels of an inhibitory NKp30 ligand on cells infected with HCV. Consequently, the impairment of NK cell-mediated cytotoxicity, ADCC, and a decline in the production of IFN-γ and TNF-α, is observed [83]. Moreover, it has been reported that HCV has the ability to activate immunosuppressive cells called myeloid-derived suppressor cells (MDSCs). This activation leads to the impairment of NK cell activity and the blocking of IFN-γ production through the action of Arginase-1 [81]. The findings from various surveys are summarized and presented in (Table 2).

**Table 2:** Alteration of phenotype and function of NK cells in virus infections

Virus	Phenotype	Outcome	Reference
Hepatitis C virus (HCV)	NKG2D downregulation	Weakened NK cell-mediated cytotoxic capability and IFN-γ production	[82]
	NKp30 downregulation but expression levels of NKG2D, NKG2A, NKp46, or CD16 remained unchanged	Inhibition of ADCC + Inhibition of IFN-γ, and TNF-α production	[83]
	NKp46 and NKp30 downregulation + NKG2A upregulation	Reduction in NCR mediated target cell killing	[84]
	NKp30 and NKp46 upregulation + increases in IL-10 production	Conserved cytotoxic activity in NK cells + influence the communication between NK cells and dendritic cells (DCs) in the liver leading to distortion of adaptive immune responses and the inability to control the virus effectively	[85]
	NKG2D and NKP30 downregulation	Reduction in NK cells’ ability to degranulate and lyse target cells and decline of IFN-γ production through direct cell-to-cell interaction between NK cells and HCV-infected hepatocytes	[86]
Hepatitis B virus (HBV)	NKG2D/DAP10 and 2B4/SAP downregulation	Weakened NK cell-mediated cytotoxic capability and IFN-γ generation	[87]
	HBV antigens leading to reduction in NKp30, NKp44, and 2B4 while,the expression of inhibitory receptor NKG2A is considerably augmented	The function of NK cells was directly hindered by HBV antigens (HBsAg and HBeAg), as they prevented NK cell activation, cytokine generation, and the release of cytotoxic granules	[88]
	CD16 and NKp30 downregulation + NKG2D upregulation + LAG3, CD274 (PD-L1), EGR2 and 3, NR4A2, and TOX upregulation + impaired mTOR	NK cell exhaustion	[78]
	Upregulation in T cell immunoglobulin- and mucin-domain containing molecule-3 (Tim-3)	Suppress NK cell functions	[89]
Human T-lymphotropic virus 1 (HTLV-1)	Declining in frequency of NKp30^+^ NK cells + CD107a upregulation (degranulation marker)	Elevated degranulation activity and production of perforin and granzyme B + Reductions in the level of NKp30 expression may impact the operational effectiveness of NK cells among individuals with HAM/TSP	[90]
	Declining in rate of CD56^+^ and CD56^dim^ cells expressing CD16 + CD107a upregulation	Decrease in NK cell mediated ADCC + elevated degranulation activity	[91]
Human immunodeficiency virus (HIV)	CD56 downregulation	Increase in dysfunctional CD56^neg^ NK cells with modified array of activating and inhibitory receptors	[92]
	Reduction in NKp30, NKp44, NKp46 and NKG2A but no change in NKG2D	Impairment of NK cell function	[93, 94]
Human papillomavirus (HPV)	Reduction in NKp30, NKp46 and NKG2D	Low NK cell cytolytic activity	[12]

Hepatitis B virus (HBV) is another virus that has the ability to induce long-lasting infection by negatively affecting the functionality of NK cells. Research findings demonstrate that during HBV infection, there is a notable decrease in the expression of NKG2D and 2B4-activating receptors on NK cells. Additionally, the presence of DAP10 and SAP, which are intracellular adaptor proteins associated with NKG2D and 2B4, respectively, is diminished. These reductions in receptor expression have a negative impact on NK cell-mediated cytotoxic capability and IFN-γ creation. Notably, greater levels of transforming growth factor-beta 1 (TGF-β1) were detected in the serum of HBV-infected patients with persistent infection. TGF-β1 is found to down-regulate the expression of NKG2D and 2B4 on NK cells, thereby impairing their effector functions. Additionally, in NK cells derived from immunotolerant patients, the expression of p15 and p21 is increased by TGF-β1, leading to the inhibition of cell-cycle progression in these cells. These findings suggest an association between TGF-β1 and the reduction in NKG2D/DAP10 and 2B4/SAP expression, leading to weakened NK cell function, which is associated with persistent HBV infection [87]. In a separate study, it was observed that in individuals with chronic HBV infection, there was a notable diminution in the levels of NK cells expressing NKp30 and 2B4 receptors. Interestingly, it has been observed that the presence of HBV antigens, such as HBsAg and/or HBeAg, can impact the expression of NK cell receptors. This effect leads to a decline in the expression of activating receptors like NKp30, NKp44, and 2B4 on NK cells. Conversely, there is a significant increase in the expression of the inhibitory receptor NKG2A resulting in the suppression of NK cell responsiveness. It is suggested that, this effect is likely mediated by the repression of STAT1, NF-κB, and p38 MAPK pathways, which are believed to be the underlying mechanisms [88]. It is worthy to note that, NK cells examined in patients with chronic hepatitis B exhibited certain characteristic molecular features commonly found in exhausted T cells. These features included elevated levels of transcription factors like TOX and NR4A-family, as well as their associated immune checkpoint targets. Furthermore, it was discovered that malfunctioning NK cells exhibited an augmentation in the expression of various fundamental genes related with T cell exhaustion. These genes include immune checkpoints and their ligands, such as LAG3 and CD274 (PD-L1), as well as transcription factors like EGR2 and 3. Additionally, it was observed that mTOR activation is hindered in NK cells obtained from patients [78].

Researches demonstrated that, NK cells could have a significant impact in halting the advancement of HTLV-1 carriers towards HTLV-1-associated myelopathy/tropical spastic paraparesis (HAM/TSP). it is found that patients diagnosed with HAM/TSP had notable decreases in the occurrence of CD56^+^CD3^−^, CD56^+^CD16^+^, CD56^+^, and CD56^dim^ cells, except for CD56^bright^ cells and CD56^+^CD3^+^ NKT cells, when compared to the frequencies observed in asymptomatic carriers (ACs) and seronegative individuals (SN). Patients with HAM/TSP also demonstrated a decline in the occurrence of CD56^+^ and CD56^dim^ cells that express CD16, the primary receptor for ADCC. Consequently, reducing the quantity of CD16 expressing cells, which play a role in ADCC against HTLV-1-infected cells, could have a substantial impact on controlling the proviral load in individuals with HAM/TSP. On the other hand, in HTLV-1-infected individuals, NK and NKT cells had significantly higher CD107a expression. CD107a is a surface marker commonly used to indicate the release of cytotoxic substances by cytotoxic cells. Also, findings indicated that there is an reverse association between the proviral load and the frequency of CD56^+^CD3^−^ cells [91]. According to another report, HTLV-1 infection can modify the quantity of activating receptors present on NK cells. In individuals diagnosed with HAM/TSP, there is a noticeable reduction in the frequency of NK cells that expressed the activating receptor NKp30 in comparison to uninfected controls. Additionally, there was an observed occurrence of elevated spontaneous degranulation in NK cells obtained from both patients with HAM/TSP and those without symptoms (AS). This degranulation led to an augmented production of granzyme B and perforin, along with the expression of CD107a and IFN-γ, contributing to the activation of lymphocytes [90]. During the early stage of HIV-1 infection, the number of NK cells in circulation undergoes an initial increase. This increase is attributed to the expansion of the CD56^dim^ NK subset and a subsequent decline in the frequency of the CD56^bright^ NK cell population [95]. In the acute phase, NK cells use several approaches to control HIV-1 infection. For instance, the production of β-chemokines by NK cells could hinder viral entrance into CD4^+^ T cells and impede the transmission of HIV-1, through stimulation with IL-2 and IL-15 [96]. As well, when NK cells identify HIV-1 infected cells, they can initiate a response by releasing IFN-γ and MIP-1β. This release of molecules has the potential to affect the body’s antiviral reaction and limit the virus’s propagation [97]. NK cells employ an extra method to restrict HIV infection, known as ADCC. It is evident that, slower disease progression has been linked to elevated levels of anti-HIV-1 antibodies that induce ADCC [98, 99]. In persistent HIV-1 infection, NK subsets undergo abnormal distribution, leading to a substantial influence on the NK cell’s antiviral ability. For instance, viral replication promotes the expansion of a CD56^−^/CD16^+^ NK cell subset that is dysfunctional (also known as anergic CD56^neg^ NK cells) [92]. Besides the reduction of CD56 on NK cell surface markers caused by HIV-1 viremia, this virus has the ability to hinder NK cell functionality by diminishing the surface expression of NKp46, NKp30, and NKp44 [93]. Although there is no significant alteration in NKG2D expression during HIV viremia, there is a downregulation of NKG2A [94]. As we know, HCMV is a significant contributor to health complications in individuals infected with HIV-1. It is linked to the upregulation NKG2C receptors. This characteristic, combined with the suppressive impact of HIV-1 on NKG2A expression, disrupts the normal NKG2A/NKG2C ratio on NK cells in patients co-infected with HIV-1 and HCMV, resulting in pathological changes [100, 101]. In addition, in HPV-associated cervical cancer and high grade squamous intraepithelial lesion patients, NK cell-activating receptors like NKp30 and NKp46 is downregulated and this leads to reduced NK cell cytotoxic activity and as well, cervical cancer patients exhibited a downregulation of NKG2D as well [12].

Altogether, to evade elimination by NK cells, viruses have established various mechanisms and approaches to counteract the immune response. These evasion tactics enable viruses to escape detection and attack by NK cells, facilitating the establishment of persistent infections in the host. The roles of NK cells in viral infections have been shown to be multifaceted, involving both protective and pathogenic functions. While these discoveries have provided valuable understanding, they also emphasize the necessity for more extensive investigations to fully comprehend the complexities of NK cell engagement in the context of this particular disease.

## The involvement of NK cells in COVID-19

Severe acute respiratory syndrome coronavirus 2 (SARS-CoV-2), which is a new type of coronavirus, had a profound impact on the world since its emergence in late 2019. It is the virus accountable for the global COVID-19 pandemic, which has affected millions of people and caused significant disruption to societies and economies worldwide. SARS-CoV-2 is a member of the coronavirus family, a group of viruses that have envelope with a single-stranded RNA genome. The entry of SARS-CoV-2 into host cells heavily relies on the vital function performed by the spike protein present on its surface. The angiotensin-converting enzyme 2 (ACE2) receptor, primarily located on the surface of respiratory epithelial cells within the lungs, is the target for the binding of the spike protein. This receptor interaction facilitates viral entry into the host cell, allowing the virus to replicate and spread throughout the body. COVID-19, the disease caused by SARS-CoV-2, displays a wide range of symptoms that can vary from mild to severe. General symptoms include fever, cough, shortness of breath, fatigue, muscle or body aches, sore throat, loss of taste or smell, and headache. In more serious instances, the infection has the potential to result in pneumonia, acute respiratory distress syndrome (ARDS), failure of vital organs, and ultimately, mortality. Certain individuals, such as the older adults and individuals with pre-existing medical conditions, face an increased likelihood of experiencing serious complications [102].

While there is still incomplete comprehension of the disease’s pathogenesis, it is believed that an overactive and misguided immune system plays a role in the advancement of severe COVID-19, potentially involving the participation of NK cells. Based on the reports, it is indicated that lymphopenia stands out as the primary characteristic indicating the seriousness of COVID-19 in patients [103]. Furthermore, numerous reports have suggested that the quantity of NK cells in the bloodstream is impacted by SARS-CoV-2 infection, with no variations in the distribution of NK cell subsets [103, 104]. It is evident that COVID-19 patients experienced a reduction in the presence of circulating NK cells, which seems to be closely associated with both the acute phase of the illness and its severity [105, 106]. As well, it has been revealed that, the rate of decrease in viral load among hospitalized patients is closely linked to the number of NK cells. Specifically, individuals with NK cell counts categorized as “normal” (> 40 cells/ml) experience a more rapid decline in viral load compared to those with “low” (≤ 40 cells/ml) NK cell numbers, irrespective of their clinical condition [107, 108]. The number of NK cells rebounds during the later phases of the illness. Conversely, individuals experiencing a fatal progression of the disease exhibit a progressive decline in NK cell levels following the onset of symptoms [103, 109]. Following recovery from COVID-19, the circulating NK cell counts in patients normalize [110]. Also, long-COVID patients displayed augmented levels of NK cells in their bloodstream compared to patients who had recovered. However, impaired virus-specific and specific effector functions were observed alongside increased levels of CD56^+^/CD57^+^/NKG2C^+^ NK cells in individuals suffering from long-COVID [111]. Altogether, these reports indicate that the measurement of circulating NK cell counts might serve as a prognostic clinical indicator for predicting the course of COVID-19. It has been proposed that the diminution in circulating NK cells may result from the relocation of these cells from bloodstream and their buildup in the lungs. Subsequently, the assessment of bronchoalveolar lavage fluids (BALFs) from COVID-19 patients through single-cell RNA sequencing (scRNA-seq) discovered augmented degrees of NK cell in the lung during the acute phase when compared to control group. This observation implies that NK cells might play a role in intensifying lung tissue damage and causing epithelial cell death [112]. It is suggested that the gathering of infected epithelial cells along with innate immune cells such as monocyte-macrophages and neutrophils may lead to the release of cytokines and chemokines. These signaling molecules subsequently attract NK cells to the lungs. This process may trigger a cytokine storm, primarily driven by IFN-γ. High levels of IFN-γ can leads ton apoptosis pf epithelial and endothelial cells, vascular leakage, and interrupt tissue homeostasis. This inflammatory condition has the capability to result in acute respiratory distress syndrome (ARDS), playing a considerable role in the morbidity and mortality linked to COVID-19 [113]. In agreement with this concept, evaluation of the serum from individuals testing positive for SARS-CoV-2 revealed a widespread inflammatory state marked by heightened concentrations of numerous pro-inflammatory cytokines. Remarkably, the levels of CXCL16, a factor related to the movement of NK cells from the bloodstream to the infected airways, seem to be augmented during the initial phase of infection in both mild and severe patients of SARS-CoV-2 [114]. Furthermore, several chemokines, namely CCL2, CCL3, CCL4, CXCL9, CXCL10, and CXCL11, display an upsurge in their expression levels within the lung tissues of COVID-19 patients. Moreover, it has been shown that NK cells in the bloodstream upregulate specific receptors, including CXCR3, CXCR6, and CCR5 in response to SARS-CoV-2 infection [112, 115, 116].

As a result, the depletion of NK cells in the peripheral blood is likely a consequence of the chemotaxis and migration of these cells towards the lungs and these cells may play a role in the cytokine storm linked to SARS-CoV-2 infection. This extreme release of cytokines is a significant contributor to the severity of the disease, principally through inflammation-induced lung damage.

Additionally, preliminary findings indicate that NK cells exhibit robust activation during acute phase of COVID-19 and display observable changes in their phenotype characteristics. Various subsets of NK cells exhibit signs of activation, as evidenced by the presence of markers such as human leukocyte antigen (HLA)-DR, CD69, and Ki-67, indicating increased expression and proliferation [106, 117]. As well, severe COVID-19 led to an upsurge in NK cells armed with heightened levels of cytotoxic proteins like perforin [106]. However, similar to other viruses, SARS-CoV-2 has the ability to employ various evasion tactics. These strategies aim to disrupt the functions of NK cells and counteract their antiviral responses. Simultaneously, alongside this, markers of exhaustion such as lymphocyte-activation gene 3 (LAG3), T cell immunoglobulin and mucin domain containing protein 3 (TIM-3), programmed cell death protein 1 (PD1), and TIGIT have been identified on NK cells in individuals diagnosed with COVID-19 [118, 119]. Ae well, COVID-19 patients exhibited decreased levels of NKG2D and DNAM-1, which are recognized as activating receptors for NK cells, when compared to individuals without the infection [120]. It has been demonstrated that patients with COVID-19 exhibit a notable elevation in the NKG2A receptor levels on their NK cells. On the contrary, Patients with COVID-19 exhibit increased HLA-E (ligand for NKG2A) expression in both their immune cells and parenchymal cells within the lungs. Thus, by engaging with HLA-E, the Spike protein-1 of SARS-CoV-2 can hinder the degranulation capacity of NK cells through the HLA-E/NKG2A pathway [121]. It is important to mention that there is an association between the emergence of adaptive-like NK cell expansions and severe/critical COVID-19. These expansions are characterized by elevated expression of NKG2C and CD57, along with restricted KIR profiles [106]. As we know, this distinct signature has primarily been examined in the context of CMV infection or reactivation. This occurrence may be attributed to various factors. It is possible that during SARS-CoV-2 infection, adaptive-like NK cells accumulate in the bloodstream due to their increased resistance to cytokine-induced apoptosis. Alternatively, the increase in adaptive-like NK cells may be prompted by SARS-CoV-2 either directly or indirectly due to the excessive generation of pro-inflammatory cytokines [122]. Moreover, the KIR haplotype is gaining recognition for its significant role in influencing the severity of diseases. For example, researches have revealed that the presence of KIR2DS5 associates with a reduced time for recovery, whereas the presence of KIR2DS2 offers protection against SARS-CoV-2 infection. Conversely, individuals carrying the KIR2DS4 and KIR2DL3 genes of the A haplotype face the greatest susceptibility to severe form of COVID-19 [123–125]. In essence, SARS-CoV-2 eventually leads to exhaustion of NK cell by elevating the expression of inhibitory receptors and declining activation receptors. This interrupts the appropriate release and communications of IFNs, ultimately compromising the antiviral abilities and immunomodulatory functions of NK cell. The exhaustion of NK cell suggests their incapacity to control overactive immune cells during the later phases of the infection, subsequently causing substantial injury to tissues, predominantly the lung tissues [111]. It is notable that the evasion mechanisms employed by SARS-CoV-2 to evade NK cells may vary from those used by other respiratory viruses including influenza. Within a few days of infection, NK cells are mobilized to the lungs, playing a vital role in the immune response against influenza. Despite this, the virus employs strategies to evade NK cell responses. First, it escapes NK cell recognition by mutating the viral HA protein. Although the interaction between NKp46 and influenza HA is a key mechanism for NK cells to identify and eliminate influenza-infected targets, the virus is adept at rapidly and frequently altering viral HA to evade antibody neutralization [126, 127]. Second, it regulates HA levels. As influenza virus replication slows down and surface HA on the epithelium or viruses becomes less abundant or inaccessible, infiltrating NK cells may not be adequately stimulated to carry out their cytolytic function [127, 128]. Finally, a particularly intriguing method involves the virus directly infecting and destroying NK cells. It is proposed that the virus has the capability to infect NK cells through clathrin- and caveolin-dependent endocytosis pathway and eventually leads to the diminution in cytotoxic activity and initiation of programmed cell death in NK cells [62, 129]. It is important to highlight that Interleukin-22 (IL-22) has gathered substantial attention in recent years, establishing itself as one of the most widely researched cytokines in the context of viral infections. Importantly, several reports have revealed that IL-22, a member of IL-10 family, plays a vital role in fighting against viral infections. It effectively mitigates immune cell-mediated inflammatory responses, lessens lung injury, and facilitates the repair and regeneration of airway epithelial tissue [130]. In the lungs, NK cells make up approximately 10% of the overall resident lymphocytes. This percentage is relatively higher than what is found in most other lymphoid and non-lymphoid tissues, suggesting the potential significant engagement of NK cells in lung infections [131]. For instance, in the initial stage of influenza infection, studies have demonstrated that NK and NKT cells in the lung display augmented expression of IL-22, leading to regeneration of airway and parenchymal epithelium.

In the sublethal stage of viral infection, IL-22 plays a role in inhibiting lung inflammation, mitigating secondary infections, and maintaining the integrity of lung epithelium. Conversely, mice lacking IL-22 and infected with the virus exhibit heightened collagen accumulation and a deficiency in epithelial regeneration [132]. Consistent with this, another study demonstrated that following influenza virus infection, lung NK cells were promptly activated, leading to the production of both IFN-γ and IL-22, along with an increased cytotoxic potential. Although the level of IL-22 in lung tissue decreased shortly after infection, gradually returning to baseline after virus clearance, the gene expression of IL-22 remained constant. Moreover, depleting NK cells, with or without influenza virus infection, resulted in a reduction of IL-22 protein levels in the lung. Surprisingly, mice treated with anti-IL-22 exhibited lower virus titers [131]. These findings suggest that during primary respiratory viral infections, IL-22 plays a marginal role in protection, indicating a distinct requirement for this cytokine in bacterial and viral infections.

Considering that COVID-19 exhibits characteristics and symptoms as other severe respiratory virus infections it is plausible to suggest that IL-22 might have a role, in reducing the severity of this respiratory disorder [130]. For instance, it becomes evident that, adult COVID-19 patients show a rise, in the numbers of Tc22 and Th22 cells that express IL-22 compared to healthy individuals, irrespective of the disease manifestation as asymptomatic pneumonia, mild pneumonia, or severe pneumonia. These observations also suggest that in the 0–12-year-old age group with an asymptomatic disease course and in uncomplicated adult cases, there is a higher presence of IL-22-expressing Tc22 cells, indicating a protective effect of IL-22 [133]. A further investigation presents evidence that SARS-CoV-2 infection is marked by the abnormal expression of interleukin-22 receptor 1 (IL22-R1) on myeloid cells in the blood and CD4^+^ T lymphocytes. The former serves as a valuable indicator for distinguishing the severity of the disease. The interaction of this receptor with various cytokines, chemokines, and cytotoxic mediators, along with its co-expression with HLA-DR high, suggests a complex mechanism that may be protective and compensatory in the early stages of infection but could potentially become harmful in later stages [134]. In a separate investigation, elevated serum levels of IL-22 and IL-33 were observed in individuals with mild/moderate COVID-19. This indicates that both cytokines might hold prognostic significance for COVID-19 and are associated with the risk of the disease [135].

Altogether, these findings suggest that NK cells experience impaired functionality, rendering them unable to halt the transmission of the disease or eliminate the infected cells and also, play a critical role in the progression of COVID-19 and other viral respiratory disease. Nevertheless, there is a pressing need for further research dedicated to understanding the involvement of NK cells in SARS-CoV-2 infection. Such studies would greatly aid in the development of effective approaches to combat SARS-CoV-2 infection. Interestingly currently, there are several clinical trials that are underway, aiming to utilize NK cells as a potential remedy for individuals afflicted with COVID-19. These trials represent a pioneering effort to explore the therapeutic potential of NK cells in combating the effects of the virus.

## NK cell deficiency and viral infection susceptibility

Congenital NK cell deficiencies are a group of rare genetic disorders characterized by defects in the function and/or number of NK cells. As we discussed, NK cells are a type of lymphocyte that play a vital role in the innate immune system’s response against infections, particularly viral infections. NK cells play a crucial role in promptly identifying and eliminating cells infected with viruses. They possess the capability to directly kill infected cells and generate antiviral cytokines, contributing to the control and resolution of viral infections. However, when NK cell function is impaired or absent due to congenital deficiencies it can result in compromised immune responses against virus, making individuals more susceptible to a range of viral infections and their associated complications [3, 136]. There are two primary categories of natural killer cell deficiency (NKDs) based on the presence of NK cells in the peripheral blood. Classical natural killer cell deficiency (cNKD) is characterized by the absence or very low presence of NK cells in the bloodstream, comprising less than 1% of the overall lymphocyte population. In contrast, functional natural killer cell deficiency (fNKD) refers to the condition where there are normal levels of NK cells in the peripheral blood; however, these NK cells exhibit impaired activity. It is important to highlight that the primary immunological deficiency causing insufficient host defense occurs in both patients with cNKD and fNKD, with the main abnormality being related to NK cells [136]. Advancements in genetic research have facilitated the identification of several genes associated with classical cNKD and fNKD (Table 3). Notably, genes such as GATA binding domain protein 2 (GATA2), maintenance complex component 4 (MCM4), minichromosomal maintenance 10 (MCM10), IFN regulatory factor 8 (IRF8), regulator of telomere elongation helicase I (RTEL1), and Go-Ichi-Ni-San (GINS) complex subunit 1 and 4 have been found to contribute to cNKD, while FCGR3A (gene encoding CD16) is associated with fNKD [137–143]. It is important to highlighted that, within these genes, MCM4, together with MCM2-7, MCM10, and GINS, constitute a strongly preserved complex that has a crucial function in both the commencement and elongation stages of DNA replication in eukaryotic organisms [144]. As further exploration continues, it is anticipated that more genetic mechanisms associated with NK cell deficiencies will be revealed. Furthermore, a significant proportion of broader primary immunodeficiency disorders (PIDs) also exhibit abnormalities in NK cell function or presence (Table 4). Individuals with NK cell deficiencies are at higher risk when facing various viral infections. One striking clinical feature of NKD is the heightened vulnerability to severe and atypical presentations of herpesviruses, such as CMV, varicella-zoster virus (VZV), HSV, EBV, and potential issues with human papillomaviruses (HPV) [136]. This emphasizes the crucial role of NK cells in the body’s defense against viral pathogens. The impact of congenital NK cell deficiencies on viral infections is not uniform and can vary depending on the specific genetic defect and the overall immune function of the affected individual. Proper diagnosis and management of these conditions are crucial to mitigate the augmented vulnerability to viral infections and their associated morbidity and mortality.

**Table 3:** Human NK cell deficiencies and subsequent viral infection susceptibility

NKD type	Gene mutation	NK cell alteration	Viral Infection susceptibility	References
cNKD	GATA2	Normal or low NK cell number + decreased NK cell lytic function + absent or severe reduction in CD56^bright^	VZV, HSV, CMV, HPV	[137, 145, 146]
	MCM4	low NK cell number + diminished CD56^dim^ subset + higher prevalence of the CD56^bright^ subset	EBV, recurrent respiratory infections	[138, 147]
	MCM10	low NK cell number + higher prevalence of the CD56^bright^ subset	fatal vulnerability to CMV	[139]
	GINS1	low NK cell number + diminished CD56^dim^ subset + higher prevalence of the CD56 ^bright^ subset	CMV	[142]
	GINS4	Reduction of NK cell number and function + severe Neutropenia	CMV, varicella, recurrent herpes simplex labialis and et al	[143]
	IRF8	low NK cell number and function + declined CD56^dim^ subset + higher prevalence of the CD56^bright^ subset	EBV	[140]
	RTEL1	severely reduced NK cell number and function	VZV	[141, 148]
fNKD	FCGR3A	Normal NK cell numbers + diminished NK cell function	EBV, HSV, HPV	[149]

**Table 4:** Several PID disorders with altered NK cell function and subsequent viral infection susceptibility

Disease	Gene mutation	NK cell alteration	Viral Infection susceptibility	References
X-linked and Autosomal recessive severe combined immunodeficiency (SCID)	IL2RG/JAK3	Absent or low NK cells	Multiple infections	[150, 151]
CD25 deficiency	IL2RA	Low NK cell number	CMV	[152]
NEMO deficiency	IKBKG	Low NK cell function	CMV	[153]
IKK2 deficiency	IKBKB	Low NK cell number	CMV	[154]
CTLA4 deficiency	CTLA4	Low NK cell function	CMV and EBV	[155]
Chediak-Higashi syndrome	LYST	Low NK cell function	EBV	[156]
X-linked lymphoproliferative syndrome type 1	SH2D1A	Low NK cell function	EBV	[157]
X-linked lymphoproliferative syndrome type 2	XIAP	Low NK cell number and function	EBV	[158]
Non-X-linked lymphoproliferative syndrome	ITK	Low NK cell number and function	EBV	[159]
Familial hemophagocytic lymphohistiocytosis 2/3/4/5	PRF1/UNC13D/STX11/STXBP2	Low NK cell function	Herpesviruses	[160–163]
Hermansky-Pudliak syndrome type 2/9	AP3BP1/BLOC1S6	Low NK cell function	Herpesviruses	[164, 165]
Wiskott-Aldrich syndrome	WAS	Low NK cell function	Herpesviruses	[166]
WIPF1 deficiency	WIPF1	Low NK cell function	Herpesviruses	[167]
RASGRP1 deficiency	RASGRP1	Low NK cell function	Herpesviruses	[168]
DOCK8 immunodeficiency syndrome	DOCK8	Low NK cell function	HPV	[169]
RLTPR deficiency	CARMIL2/RLTPR	Low NK cell function	HPV	[170]
X-linked hyper IgM	CD40LG	Low NK cell function	Enteroviruses	[171]

## Future perspective

NK cells are crucial components of the body’s immune system and are actively involved in protecting against a range of illnesses, such as cancer and viral infections. As part of the initial immune response, NK cells promptly identify and eliminate target cells without requiring prior exposure to antigens or being restricted by MHC molecules. This unique ability makes them valuable candidates for immunotherapeutic strategies aimed at combating diseases [172]. The field of cancer treatment has been transformed by immunotherapy, which utilizes the immune system’s potential to specifically attack and eradicate cancerous cells. Among the diverse array of immune cells, NK cells have emerged as a talented player in immunotherapeutic strategies. In recent years, researchers have explored different modes of utilizing NK cells in immunotherapy, aiming to enhance their antitumor activity and broaden their therapeutic potential. These approaches encompass a range of techniques, including adoptive cell therapy (ACT) of NK cells, genetic engineering to improve NK cell specificity and functionality, cytokine therapy, monoclonal antibody therapy, and combination therapies with other immune-modulating agents [172, 173]. The utilization of NK cells in ACT constitutes a significant approach within the field of NK cell immunotherapy. This approach involves isolating NK cells from a patient’s peripheral blood, induced pluripotent stem cells (iPSCs) cord blood, or NK cell lines and culturing them with cytokines and activating agents to promote their proliferation and enhance their cytotoxicity. The expanded NK cells can then be infused back into the patient, bolstering the immune response against cancer cells [174]. In the realm of NK cell therapy for cancer patients, various genetic engineering methods have emerged to enhance the anti-tumor activity of NK cells and improve adoptive NK cell therapy. The primary focus of genetic engineering in NK cells revolves around equipping them with chimeric antigen receptors (CARs) or other genetic constructs. Similar to CAR-T cell therapy, CAR NK cells are altered to express a synthetic receptor that recognizes specific tumor-associated antigens, thereby enhancing their targeting capabilities [175]. Alternative genetic modifications that do not rely on CAR structures have been investigated to boost the cytotoxicity and in vivo persistence of NK cells. One such modification involves introducing high-affinity non-ADAM17 cleavable CD16 (hnCD16) or CD64, which considerably improves the binding affinity of NK cells to tumor-targeting antibodies. This, in turn, improves ADCC [176]. In addition to enhancing cytotoxicity, strategies have been developed to develop the in vivo persistence of NK cells. One approach involves maintaining cell viability and quantity by deleting the CISH gene or expressing c-MPL or EPOR, which help support NK cell survival under low cytokine concentrations [177, 178]. Another method to prolong NK cell persistence is through the deletion of endogenous CD38 or MHC-I molecules [173]. These genetic modifications offer potential ways to augment NK cell function and increase their persistence within the body, ultimately improving their effectiveness in targeting tumors and mediating immune responses. Continued research in these areas holds promise for advancing NK cell-based immunotherapies and their clinical applications. An alternative method involves infusing patients with cytokines like IL-2 and IL-15 to stimulate the activity of their endogenous NK cells in the body [179]. However, despite their promise, NK cell therapy faces challenges including the partial persistence and survival of transferred NK cells within the hostile tumor microenvironment. Moreover, NK cells may experience exhaustion as a result of extended stimulation or exposure to inhibitory signals within tumor microenvironments or during chronic infections. This exhaustion hampers their antitumor activity, compromising the efficacy of NK cell-based therapies [180]. To address this limitation, researchers are exploring the usage of monoclonal antibodies as a potential solution to reduce NK cell exhaustion. In the context of NK cell therapy, monoclonal antibodies (mAbs) can be employed to modulate inhibitory receptors and restore NK cell function. For example, certain antibodies like Monalizumab directly target NK cell inhibitory receptors, such as NKG2A, stimulating NK cells to effectively eliminate tumor cells [181]. Additionally, Lirilumab acts as an inhibitor of inhibitory KIR receptors, specifically binding to KIR2DL1, -2, and -3 receptors with strong affinity, while also activating KIR2DS1 and -2 receptors. Lirilumab promotes the destruction of tumor cells by enhancing the ability of NK cells to kill them through the inhibition of the interaction between KIR and HLA-C [182]. Additionally, NK cells express co-inhibitory receptors such as TIGIT, LAG-3, and TIM-3, which contribute to regulating their activity [183]. Ligands of these receptors are abundantly present on cancer cells and have been associated with exhaustion of NK cells and unfavorable prognosis. Consequently, hindering these receptors can prevent the exhaustion of NK cells that infiltrate cancerous tissues and promote a robust anti-cancer response. Moreover, the inhibition of PD-1/PD-L1 signaling via mAbs such as Nivolumab and Pembrolizuma can significantly amplify the anticancer potency of NK cells and impede the advancement of cancer [184]. For example, in the phase II experiment, the combination of the TIGIT antibody tiragolumab and the PD-L1 antibody atezolizumab demonstrated a notable advantage in the treatment of non-small cell lung cancer (NSCLC) patients with high PD-L1 expression [185]. It is crucial to emphasize that, ADCC plays a crucial role in NK cell anti-tumor function, facilitated by the interaction between CD16 and IgG. The activation of NK cells can be attained through the binding of the CD16 receptor by IgG monoclonal antibodies. Several monoclonal antibodies, such as trastuzumab, pertuzumab, cetuximab, rituximab, and ofatumumab, have been associated with promoting ADCC [184].

In conclusion, the different modes of immunotherapeutic applications of NK cells offer diverse approaches to harness the potential of these innate immune cells. Ex vivo expansion, genetic engineering, and combination therapies provide avenues to enhance NK cell function, specificity, and persistence, thereby improving their efficacy in targeting cancer cells and combating viral infections. Harnessing the potent antiviral properties of NK cells could lead to the design of immunotherapeutic strategies that enhance NK cell function and promote viral clearance. Additionally, understanding the mechanisms employed by viruses to elude NK cell surveillance can guide the development of targeted interventions to overcome these immune evasion tactics. Altogether, the dynamic and intricate relationship between NK cells and viral infections holds great promise for advancing our understanding of host-virus interactions and developing innovative approaches for the prohibition and treatment of viral diseases. Continued research in this field is vital to unlock the full therapeutic potential of NK cells and pave the way for improved clinical outcomes in the fight against viral infections.

## Data Availability

Not applicable.
